# Elevated Netrin-4 Expression and Its Action in Infrapatellar Fat Pad

**DOI:** 10.3390/ijms252111369

**Published:** 2024-10-22

**Authors:** Yui Uekusa, Manabu Mukai, Ayumi Tsukada, Dai Iwase, Jun Aikawa, Naoya Shibata, Yoshihisa Ohashi, Gen Inoue, Masashi Takaso, Kentaro Uchida

**Affiliations:** 1 Department of Orthopedic Surgery, School of Medicine, Kitasato University, 1-15-1 Minami-ku Kitasato, Sagamihara City 252-0374, Kanagawa, Japan; uekusa.yui@st.kitasato-u.ac.jp (Y.U.); m.manabu0829@hotmail.co.jp (M.M.); amidesutarere9010@yahoo.co.jp (A.T.); daiiwase19760601@yahoo.co.jp (D.I.); jun43814@gmail.com (J.A.); shibachoku999@gmail.com (N.S.); 44134413oo@gmail.com (Y.O.); ginoue@kitasato-u.ac.jp (G.I.); mtakaso@kitasato-u.ac.jp (M.T.); 2 Medical Sciences Research Institute, Shonan University, Nishikubo 500, Chigasaki City 253-0083, Kanagawa, Japan

**Keywords:** Netrin-4, osteoarthritis, infrapatellar fat pad

## Abstract

Knee osteoarthritis (KOA) is a degenerative joint disease characterized by inflammation and cartilage degradation. The infrapatellar fat pad (IFP), located beneath the patella within the knee joint, serves as a key anatomical structure involved in cushioning and supporting the knee. It is also an active endocrine organ that secretes various bioactive substances, potentially influencing the local inflammatory environment and contributing to KOA pathogenesis. Netrin-4 (NTN4), a protein primarily known for its role in neuronal guidance, has been implicated in various non-neuronal functions, including inflammatory processes and tissue remodeling. This study aims to explore the involvement of NTN4 in KOA, focusing on its expression in the IFP and its potential impact on disease progression. This study involved 82 patients with radiographically confirmed KOA undergoing total knee arthroplasty (TKA). The correlation between *NTN4* expression and OA pathology, including Kellgren–Lawrence (K/L) grades, was investigated. *NTN4*-expressing cells were identified in the stromal vascular fraction, including fibroblastic, hematopoietic, and endothelial cells of the IFP. To elucidate the molecular effects of NTN4, RNA sequencing (RNA-seq) was performed on fibroblastic cells treated with recombinant NTN4. Subsequent quantitative PCR (qPCR) was used to validate the RNA-seq findings. *NTN4* expression was significantly elevated in the IFP of patients with advanced KOA (K/L grades 3 and 4) compared to those with early-stage disease (K/L grade 2). Higher *NTN4* expression was found in fibroblastic cells, and RNA-seq analysis revealed upregulation of genes associated with pro-inflammatory pathways, including IL-17 and TNF-α signaling, and matrix degradation. Notably, genes including *IL6*, *MMP1*, *CXCL1*, and *CXCL8* were significantly elevated, as confirmed by qPCR, indicating NTN4’s role in promoting an inflammatory and catabolic environment. Our findings suggest that NTN4 plays a significant role in the pathogenesis of KOA by promoting inflammation and matrix degradation within the IFP. Although *NTN4* expression was not directly correlated with clinical symptoms, its elevated expression in fibroblastic cells and influence on inflammatory and degradative pathways suggest a potential mechanism for exacerbating joint damage. Targeting NTN4 could offer a novel therapeutic approach to mitigating inflammation and slowing disease progression in KOA, ultimately improving patient outcomes. Further research is needed to clarify NTN4’s specific roles and therapeutic potential in OA management.

## 1. Introduction

Osteoarthritis (OA) is the most common joint disease, characterized by abnormal remodeling of joint tissues influenced by inflammatory mediators. It is a leading cause of disability among older adults, significantly affecting quality of life due to pain and reduced mobility [1,2]. This chronic condition impacts several components within the joint, including cartilage, subchondral bone, synovium, and ligaments, as well as structures outside the joint, such as muscles, bursae, nerves, and peri-articular fat pads.

Due to the intra-articular yet extrasynovial location of Hoffa’s fat pad or infrapatellar fat pad (IFP) within the knee joint, there is increasing interest in its potential role in the development of knee osteoarthritis (KOA) [3]. The IFP comprises a fibrous framework embedded with adipose tissue, which is responsible for distributing synovial fluid and mechanical forces within the knee [3,4]. It functions as a morphofunctional unit alongside the synovial membrane, which secretes various cytokines, interleukins (ILs), adipokines, and growth factors, many of which influence synovial fluid levels [3,5,6,7,8,9,10,11]. However, the molecular mechanisms within the IFP are not yet fully understood.

Netrin class proteins are part of the larger family of neuronal guidance cues, initially discovered in the nervous system for their role in directing axon growth. However, they were later found to be expressed and functional in various other tissues [12]. Among the netrin family proteins, netrin-4 (NTN4) has been identified in tumor cells, fibroblastic cells, and endothelial cells [13,14,15]. NTN4 is a unique member of the netrin family for several reasons. Firstly, its N-terminal domains are homologous to the laminin β chain, while those of other secreted netrins are homologous to the laminin γ chain [12]. Secondly, NTN4 has a low binding affinity for the canonical netrin receptors UNC5B, NEO1, and DCC [16]. Instead, NTN4 binds to the laminin γ chain in a manner that can disassemble the laminin network [16,17].

The role of NTN4 in inflammation and disease is an emerging area of research. In cancer, for example, NTN4 has been shown to contribute to tumor progression by modulating the tumor microenvironment [18,19]. It also influences angiogenesis, a critical process for tumor growth, by interacting with endothelial cells and promoting the formation of new blood vessels [20]. Additionally, studies indicate that NTN4 can exacerbate inflammatory responses under certain conditions. A previous study indicated that NTN4 contributes to endothelial inflammation [21]. Additionally, a recent study reported that NTN4 expression in synovial fibroblast derived from rheumatoid arthritis patients correlated with pain [22]. Given its involvement in these processes, NTN4 represents a potential biomarker and therapeutic target in various diseases. In OA, where inflammation and tissue degradation are central to disease progression, understanding the role of NTN4 could provide new insights into disease mechanisms and treatment strategies The potential involvement of NTN4 in KOA has not been extensively studied, particularly in the context of the IFP. The IFP’s unique position within the knee joint and its role as a source of inflammatory mediators make it a critical area of interest for understanding OA pathogenesis. Given NTN4’s known functions in inflammation and angiogenesis, we hypothesize that NTN4 may be upregulated in the IFP of KOA patients and contribute to disease progression.

Here, for the first time, we have provided supporting evidence for relationship between NTN4 in IFP and OA. In this study, association between *NTN4* mRNA in IFP and radiographic OA grade was evaluated by quantitative PCR analysis. In addition, cell types expressing *NTN4* were investigated in IFP. Meanwhile, gene alterations, molecular functions, and regulation pathways of *NTN4* were explored. Our findings shed light on the role of NTN4 in OA and provided potential interaction between NTN4 and OA.

## 2. Results

### 2.1. Correlation Between Proportion of NTN4 Expression and OA Pathology

Table 1 presents the demographic and clinical characteristics of the 82 patients included in the study. The cohort consisted of individuals with varying Kellgren–Lawrence (K/L) grades, representing different stages of osteoarthritis (OA) severity. Clinical features such as age, BMI, pain at rest (VAS-R), and pain on movement (VAS-M) were also recorded (Table 1).

**Figure 1:**
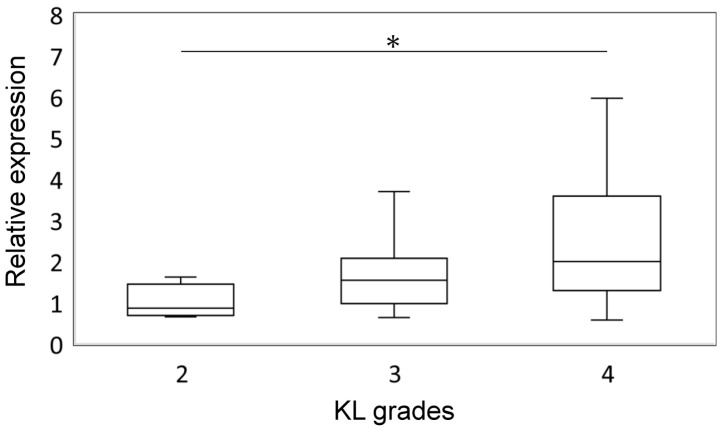
Relationship between *NTN4* expression and radiographic OA grades. * indicates a significant difference compared to the KL2 group (*p* < 0.05).

To elucidate the relationship between *NTN4* expression and the severity of KOA, we analyzed the expression levels of *NTN4* in the IFP of patients categorized by different K/L grades. Specifically, we examined samples from individuals with K/L grades 2, 3, and 4, representing increasing severity of OA. qPCR analysis revealed that *NTN4* expression was 1.75-fold higher in individuals with K/L grade 3 compared to those with K/L grade 2, although this increase was not statistically significant (*p* = 0.411). In contrast, *NTN4* expression was significantly elevated in patients with K/L grade 4, showing a 2.46-fold increase relative to those with K/L grade 2 (*p* = 0.040) (Figure 1). These findings suggest a potential association between higher *NTN4* expression and more advanced OA, indicating that NTN4 may play a role in the progression of joint degeneration.

To ensure that the differences in *NTN4* expression were not influenced by potential confounders, we performed an Analysis of Covariance (ANCOVA) to adjust for age, sex, BMI, pain at rest, and pain on movement. The results indicated that none of these variables significantly affected *NTN4* expression (age, *p* = 0.591; sex, *p* = 0.058; BMI, *p* = 0.154; VAS-R, *p* = 0.580; VAS-M, *p* = 0.795), except for K/L grade, which showed a significant association with *NTN4* expression (*p* = 0.027, Table 2). Therefore, these factors were not considered confounders, and the observed differences in *NTN4* expression across K/L grades are likely related to the severity of osteoarthritis itself.

Additionally, we conducted a linear regression analysis to explore the relationship between *NTN4* expression and key clinical variables such as K/L grade, age, BMI, VAS-R, and VAS-M. The results showed that KL grade was significantly associated with *NTN4* expression (β = 0.309, *p* = 0.007), indicating that higher KL grades are positively correlated with increased *NTN4* expression levels. In contrast, age (β = −0.059, *p* = 0.591), Sex (β = −0.211, *p* = 0.057), BMI (β = 0.156, *p* = 0.147), VAS-R (β = −0.066, *p* = 0.586), and VAS-M (β = −0.030, *p* = 0.805) were not significantly associated with *NTN4* expression (Table 3). These findings suggest that *NTN4* expression is primarily influenced by OA severity, as represented by KL grade, while age, BMI, and pain scores do not have a significant impact on *NTN4* expression in this cohort.

**Figure 2:**
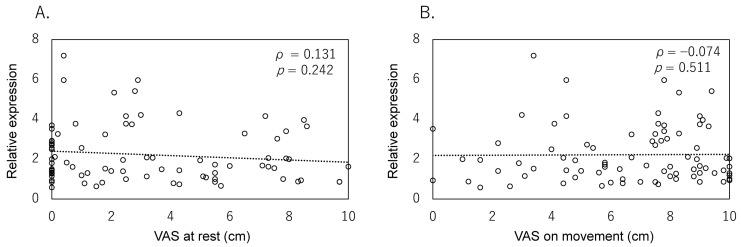
Relationship between *NTN4* expression and pain score. Correlation analysis between *NTN4* expression levels and pain scores, as measured by the Visual Analog Scale (VAS) at rest (VAS-R) (**A**) and on movement (VAS-M) (**B**).

Finally, we assessed whether *NTN4* expression correlates with clinical symptoms, particularly pain. Pain was assessed using the VAS for both VAS-R and VAS-M. Statistical analysis showed no significant correlation between *NTN4* expression and VAS-R (ρ = 0.131, *p* = 0.242) or VAS-M (ρ = −0.074, *p* = 0.511) (Figure 2A,B). These results suggest that while *NTN4* expression increases with OA severity, it may not directly influence pain perception in patients.

### 2.2. NTN4-Expressing Cells in SVF

**Figure 3:**
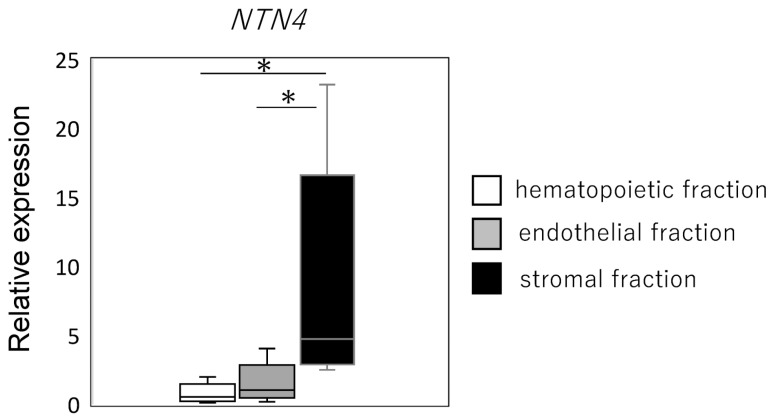
*NTN4* expression in stromal vascular fractions. Expression levels of *NTN4* in hematopoietic, endothelial, and stromal cell fractions are derived from the infrapatellar fat pad (IFP). * indicates significant differences between groups (*p* < 0.05).

Given the observed elevation of *NTN4* expression in patients with severe OA (K/L grade 4), we sought to identify the specific cell populations within the IFP that express *NTN4*. The IFP samples were processed to isolate the SVF, which was subsequently divided into HF, EF, and FF cell fractions. qPCR analysis revealed that *NTN4* expression was significantly higher in the FF compared to both the HF and the EF. Specifically, the FF exhibited a marked increase in *NTN4* expression compared to HF (*p* < 0.001) and EF (*p* = 0.013) (Figure 3). There was no significant difference in *NTN4* expression between HF and EF (*p* = 0.307). The high expression of *NTN4* in fibroblastic cells suggests that these cells may be a primary source of NTN4 in the IFP.

### 2.3. Effect of NTN4 on IFP-Derived Fibroblastic Cells

**Figure 4:**
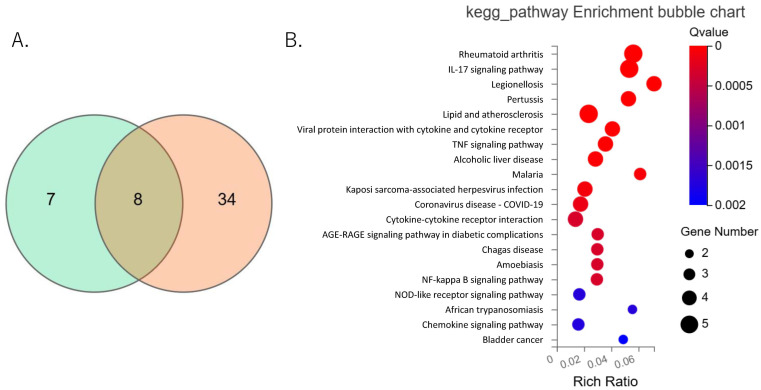
RNA-sequencing of vehicle and recombinant Netrin-4 treated fibroblastic cells derived from the IFP. (**A**) Common upregulated genes in Netrin-4 (NTN4)-treated cells compared to vehicle-treated cells from two patient-derived cell samples were identified and visualized using a Venn diagram. (**B**) KEGG pathway analysis of the commonly upregulated genes in NTN4-treated cells.

To explore the functional impact of NTN4 on IFP-derived fibroblastic cells, we conducted a differential gene expression (DEG) analysis using RNA sequencing (RNA-seq). Fibroblastic cells isolated from IFP were treated with either vehicle or 500 ng/mL recombinant NTN4. The RNA-seq analysis identified eight genes that were significantly differentially expressed between the NTN4-treated and vehicle-treated groups (Table 4, Figure 4A). Notably, KEGG pathway analysis indicated enrichment in pathways associated with rheumatoid arthritis, IL-17 signaling, TNF-α signaling, and NF-kappa B signaling (Figure 4B). These pathways are well-known for their roles in mediating inflammatory and immune responses, suggesting that NTN4 may influence these processes in the context of OA.

**Figure 5:**
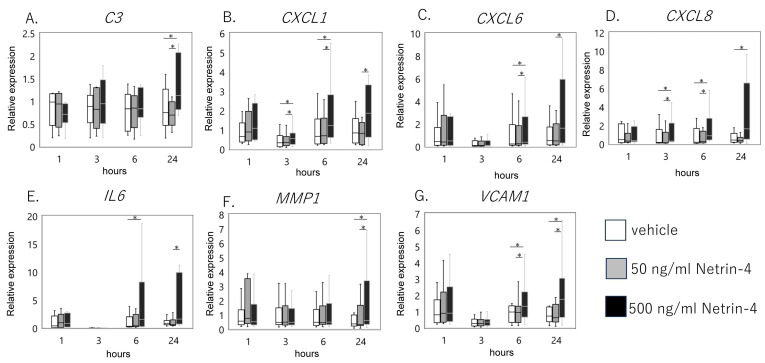
qPCR analysis of vehicle and recombinant Netrin-4 treated fibroblastic cells derived from the IFP. (**A**) *C3* (**B**) *CXCL1*, (**C**) *CXCL6*, (**D**) *CXCL8*, (**E**) *IL6*, (**F**) *MMP1*, and (**G**) *VCAM1*. * indicates significant differences between groups at each time point (*p* < 0.05).

To validate and further explore these findings, we examined the expression levels of key genes within these pathways at various time points and NTN4 concentrations. Upon stimulation with 500 ng/mL NTN4, the complement component 3 (*C3*) gene exhibited a significant increase in expression after 24 h (Figure 5A, *p* = 0.042). The chemokine (C-X-C motif) ligand 1 (*CXCL1*) showed a significant upregulation at 3 h (*p* = 0.011), 6 h (*p* < 0.001), and 24 h (*p* = 0.011) post-treatment (Figure 5B). Similarly, *CXCL6* expression was significantly elevated after 6 h (Figure 5C, *p* = 0.011), while *CXCL8* levels increased significantly at 3 h (*p* = 0.011), 6 h (*p* = 0.022), and 24 h (Figure 5D, *p* = 0.022). Interleukin-6 (*IL6*), a key pro-inflammatory cytokine, also showed significant upregulation at 6 h (*p* = 0.001) and 24 h (*p* = 0.042) following NTN4 treatment (Figure 5E). Matrix metalloproteinase 1 (*MMP1*), an enzyme involved in collagen degradation, was significantly upregulated after 24 h (Figure 5F, *p* = 0.007), indicating a potential role in matrix remodeling and degradation. Moreover, vascular cell adhesion molecule 1 (*VCAM1*) expression increased significantly at 6 (*p* = 0.005) and 24 (*p* = 0.022) hours (Figure 5G), suggesting enhanced cell adhesion and potential immune cell recruitment. Lower concentrations of NTN4 (50 ng/mL) did not result in significant changes in the expression levels of these genes compared to the vehicle control. This dose-dependent response indicates that higher levels of NTN4 are required to exert significant biological effects on fibroblastic cells. These findings collectively suggest that NTN4 acts as a modulator of inflammatory and catabolic responses in IFP-derived fibroblastic cells.

## 3. Discussion

This study found that *NTN4* expression was elevated in the IFP of patients with severe radiographic KOA. Higher *NTN4* expression was detected specifically in fibroblastic cell fractions. Furthermore, NTN4 was observed to stimulate the production of IL-6, MMP1, and various chemokine ligands, suggesting that NTN4 in the IFP may contribute to the progression of OA through mechanisms involving inflammation, immune cell infiltration, and matrix degradation.

Previous studies have established the IFP’s role in knee pain perception due to its nerve fiber endings [23,24]. NTN4, known for its role in axonal outgrowth and neuronal branching, has been shown to promote neurite growth in cultured olfactory bulb explants and increase branching of pain-sensitive neurons in murine models [20,22]. Our findings, however, did not show a direct correlation between *NTN4* expression and pain perception. Instead, higher *NTN4* expression was associated with late-stage OA. This discrepancy suggests that while NTN4 may influence neuronal growth and branching, its role in pain perception may be more complex and potentially involves other mediators or pathways not captured in our study.

IFP consist of hematopoietic cells (lymphocytes, macrophages, mast cells), adipocyte, endothelial cells, and stromal cells. Previous study reported that NTN4 is abundantly expressed by lining fibroblast in synovium derived from rheumatoid arthritis patients [22]. However, its expression in the IFP has remained unclear. Our findings demonstrate that fibroblastic cells within the IFP exhibit high levels of *NTN4* expression, suggesting that NTN4 is primarily produced by these fibroblastic cells in the IFP.

The interplay between the IFP and cartilage metabolism is well-documented, with previous research indicating that media conditioned by IFP from osteoarthritic knees exerts pro-catabolic effects on cartilage explants and chondrocytes [25]. Our study extends these findings by showing that NTN4 stimulates *MMP1* expression in IFP-derived fibroblastic cells, supporting its role in cartilage degradation. This aligns with the established role of MMP-1 and MMP-13 in OA, where they act as rate-limiting enzymes in collagen degradation [26]. The increased expression of MMPs in OA-derived stromal cells compared to those from patients with anterior cruciate ligament tears [27] further underscores the catabolic environment promoted by NTN4 in OA progression.

Our findings highlight the IFP as a site of immune cell infiltration and a source of pro-inflammatory mediators, consistent with previous studies that have identified increased levels of TNF-α, IL-1, IL-6, and IL-8 in the IFP of OA patients [28,29,30]. Recent studies have also reported an increase in CD14++CD80+ and CD14++CD163+ cells in the IFP of end-stage OA patients, contributing to local inflammation [29]. We observed that NTN4 promotes the expression of chemokine ligands (*CXCL1*, *CXCL6*, *CXCL8*), which recruit myeloid cells and enhance the inflammatory milieu within the IFP. This is consistent with studies in other diseases, such as breast cancer, where *NTN4* expression was positively associated with immune cell infiltration [31]. Our data suggest that NTN4 exacerbates inflammation in the IFP by promoting immune cell recruitment, thereby contributing to the OA.

Our study contributes to the existing body of research by providing a comprehensive analysis of NTN4’s role in the IFP of KOA patients. While previous research has highlighted NTN4’s involvement in pain perception, our findings suggest a more nuanced role. NTN4 appears to primarily drive OA progression through inflammation and matrix degradation rather than directly influencing pain. This distinction is critical for developing targeted therapeutic strategies aimed at modulating NTN4 activity to slow or halt OA progression without necessarily focusing on pain relief.

Future research should explore the complex interactions between NTN4 and other inflammatory mediators, as well as its potential synergistic effects with other pathways involved in OA. Additionally, investigating the mechanisms underlying NTN4’s differential effects on pain perception and OA progression could provide valuable insights for therapeutic interventions. Understanding the broader systemic effects of NTN4, beyond the local environment of the IFP, will also be crucial in developing comprehensive treatment strategies for OA.

This study has several limitations that may affect the generalizability of our findings. Firstly, the sample was derived from a single institution, and all participants were undergoing TKA for KOA. As such, the results may not be fully representative of broader KOA populations, including those in earlier stages of the disease or those receiving other forms of treatment. Additionally, the study did not include a control group of healthy individuals, limiting our ability to compare *NTN4* expression between osteoarthritic and non-osteoarthritic conditions.

Secondly, the relatively small sample size may affect the statistical power to detect smaller, but potentially clinically relevant, differences in *NTN4* expression. We did not perform a formal sample size calculation, and future studies should incorporate power analyses to ensure that adequate sample sizes are used to improve the robustness and generalizability of the findings. Thirdly, the CD45–CD31– fraction, which we identified as containing stromal cells, includes a heterogeneous mix of fibroblasts, mesenchymal stem cells, adipocyte precursors, and preadipocytes. Further investigation is needed to delineate the specific contributions of these subpopulations to NTN4 production and OA pathology. Future studies should aim to include a more diverse patient population from multiple institutions, include healthy control groups, and utilize larger sample sizes to enhance the generalizability of the results to broader populations.

In conclusion, this study provides compelling evidence that NTN4 plays a pivotal role in the progression of knee osteoarthritis through its contributions to inflammation, immune cell recruitment, and matrix degradation within the infrapatellar fat pad. While NTN4’s direct role in pain perception remains unclear, its involvement in key inflammatory and catabolic pathways presents a promising target for future therapeutic interventions. Targeting NTN4 could represent a novel approach in OA treatment, not only to mitigate inflammation but potentially to slow disease progression and protect joint integrity. The broader implications of these findings suggest that modulating NTN4 activity could open new avenues for treatment strategies in OA that go beyond symptomatic relief, potentially addressing the underlying molecular drivers of the disease. Future research exploring small molecule inhibitors, biologics, or gene therapies targeting NTN4 may offer innovative ways to improve patient outcomes, reduce joint degradation, and possibly delay or prevent the need for surgical interventions like total knee arthroplasty. Further investigation into NTN4’s interactions with other inflammatory mediators and its systemic effects will be crucial in translating these findings into clinical applications.

## 4. Materials and Methods

### 4.1. Patients and Methods

This retrospective, single-institution study was conducted according to the principles of the Declaration of Helsinki, and the study protocol was approved by the Institutional Review Board of Kitasato University (Approval number, B19-259). All data were anonymized. The institutional review board waived the requirement for written informed consent because of the retrospective study design, as medical records and archived samples were used with no risk to the participants. We applied Opt-out method to obtain consent on this study. The study included 82 patients diagnosed with knee osteoarthritis (KOA) who underwent total knee arthroplasty (TKA) at Kitasato University Hospital. Patients were selected based on the availability of complete medical records and sufficient tissue samples for analysis. Inclusion criteria consisted of a diagnosis of KOA, confirmed by clinical and radiographic evaluations, and a willingness to contribute anonymized data and tissue samples. Exclusion criteria included a history of other inflammatory joint diseases, prior knee surgeries, or recent intra-articular injections, which could confound the study results.

Radiographic severity of KOA was assessed using the Kellgren-Lawrence (K/L) grading system, a widely accepted method for evaluating osteoarthritis severity based on joint space narrowing, osteophyte formation, and subchondral sclerosis observed in radiographs. The grades range from 0 (no radiographic features of OA) to 4 (severe OA). Pain levels were measured using the Visual Analog Scale (VAS) at rest (VAS-R) and on movement (VAS-M). The VAS is a 10-point scale where 0 represents no pain, and 100 represents the worst imaginable pain.

### 4.2. NTN4 Expressing Cells in Stromal-Vascular Fraction of IFP

To investigate the *NTN4*-expressing cells in the stromal vascular fraction (SVF), which is the component of adipose tissue that includes a heterogeneous mixture of cells such as fibroblasts, endothelial cells, and immune cells, we used magnetic bead methods to isolate hematopoietic cells, endothelial cells, and fibroblastic cells from the IFP. Fresh IFP samples were immediately immersed in a collagenase solution at a concentration of 400 U/mL for 2 h at 37 °C to facilitate digestion. The cells were then incubated for 30 min at 4 °C with PE-conjugated anti-CD45 (a pan-leukocyte marker) (BioLegend, San Diego, CA, USA). After two washes with phosphate-buffered saline (PBS), the cells were treated with anti-PE magnetic particles (BD Biosciences, Milpitas, CA, USA) and introduced into a magnetic separation system (BD Biosciences) to separate them into a negative fraction (enriched with non-hematopoietic cells) and a positive fraction (hematopoietic cell-rich fraction, HF). Next, the non-hematopoietic cell-rich fraction was incubated for 30 min at 4 °C with a biotin-conjugated anti-CD31 antibody (an endothelial cell marker, eBiosciences, cat. no. 13-0319-82, Thermo Fisher SCIENTIFIC, Waltham, MA, USA). After two PBS washes, the cells were treated with streptavidin-conjugated magnetic particles (BD Biosciences, CA, USA) to segregate them into a CD31-positive fraction (endothelial cell-rich fraction, EF) and a CD31-negative fraction (fibroblastic cell-rich fraction, FF). HF, EF, and FF were then subjected to qPCR to examine *NTN4* expression. Relative mRNA expression was determined using the mean value calculated for the HF as the reference.

### 4.3. Effect of Netrin-4 Protein in Stromal Cells

Next, to investigate the effect of NTN4 on fibroblastic cells of the IFP, the cells were incubated for 30 min at 4 °C with biotin-conjugated anti-CD31 and CD45 antibodies following the collagenase digestion described above. After two washes with phosphate-buffered saline (PBS), the cells were treated with streptavidin-conjugated magnetic particles and introduced into a magnetic separation system (BD Biosciences) to isolate the FF. The cell suspension was then transferred to a 75 cm^2^ culture flask. SCs were maintained in a humidified incubator at 37 °C with 5% CO_2_, and the culture medium, α-minimal essential media (α-MEM; Nacalai Tesque Inc., Kyoto, Japan) containing 10% fetal bovine serum, was changed every third or fourth day. Subconfluent SCs were detached using a 0.25% trypsin/EDTA solution and seeded at 2 × 10^5^ cells per well in a 6-well plate. After 3 days, the SCs were incubated with either the vehicle (culture medium) or 50 or 500 ng/mL human recombinant NTN4 (R&D systems, Minneapolis, MN, USA) for 1, 3, 6, and 24 h. Following incubation, the cells were lysed in TRIzol solution, and total RNA was purified for subsequent RNA sequencing (*n* = 2) and qPCR analysis (*n* = 10).

### 4.4. RNA-Sequencing

To identify genes with differential expression between vehicle and 500 ng/mL NTN4, SCs from 2 patients, RNA-sequencing RNA quantity was measured with a spectrophotometer (Denovix, Wilmington, DE, USA), and RNA quality was assessed using an Agilent 2100 BioAnalyzer (Agilent Technologies, Santa Clara, CA, USA) with an RNA 6000 Nano Chip. The isolated RNA then underwent RNA-seq analysis on the DNBSEQ Technology Platform before being sent to BGI Japan for further RNA-seq analysis. Differential gene expression was identified using specific criteria in the RNA-seq analysis, where genes with a false discovery rate (FDR) ≤ 0.001 and a log2 fold change ≥ 1 were considered significant [32]. Pathway analysis was conducted using the Kyoto Encyclopedia of Genes and Genomes (KEGG; http://www.genome.jp/kegg/, accessed on 15 August 2024) to explore significant pathways. To validate the results obtained through RNA-Seq analysis, we conducted qRT-PCR targeting a DEFs from ten patients.

### 4.5. qPCR

To validate the RNA-seq results, qPCR was performed on a subset of DEGs identified in the RNA-seq analysis. RNA samples from the FF of additional patients (*n* = 10) were subjected to qPCR. Synovial tissue and cell samples were homogenized in TRIzol reagent (Invitrogen, Carlsbad, CA, USA) using a polytron homogenizer. After homogenization, the samples were lysed in a mixture of 1 mL TRIzol and 0.2 mL chloroform, vortexed for 30 s, and then transferred to a MaXtract high-density tube (Qiagen, Valencia, CA, USA). Following centrifugation (18,000× *g*, 4 °C, 5 min), the resulting aqueous phase was combined with an equal volume of isopropanol containing a precipitation carrier (Ethachinmate; Nippon Gene, Tokyo, Japan). The RNA pellet was obtained after removing the supernatant, washed with 70% ethanol, and centrifuged again (18,000× *g*, 4 °C, 5 min). The supernatant was discarded, and the RNA pellet was air-dried and dissolved in RNase-free water. The total RNA concentration was measured using a spectrophotometer (Denovix, Tokyo, Japan), with an OD 260/280 ratio greater than 1.8 deemed suitable for qPCR analysis. Primer sequences for are provided in Table 5. The primer sequences were determined using Primer-BLAST software (National Center for Biotechnology Information, Bethesda, MD, USA; https://www.ncbi.nlm.nih.gov/tools/primer-blast/) (accessed on 5 March 2021), and all primers used were oligonucleotide purification cartridge-purified and obtained from Hokkaido System Science (Sapporo, Japan). Synovium samples was obtained from OA patients during total knee arthroplasty. Gene expression (Gene/*GAPDH*) was determined using the delta-delta CT method, with relative expression calculated based on the average gene expression (Gene/*GAPDH*) level in the Kellgren–Lawrence (KL) grade 2 (KL2) or vehicle group set to 1.

### 4.6. Statistical Analyses

Statistical analyses were conducted using SPSS software (Version 28.0; IBM Corp., Armonk, NY, USA). Initially, the Shapiro–Wilk test was applied to assess the normality of the data distributions for each group. Given that the data did not meet the assumption of normality (*p* < 0.05 in Shapiro–Wilk test), non-parametric statistical methods were employed. Differences among groups were assessed using the Kruskal–Wallis test, a non-parametric equivalent of one-way ANOVA, which does not assume normal distribution of data. This test was used to compare *NTN4* expression levels across different K/L grades and to analyze the effects of NTN4 treatment on gene expression levels in fibroblastic cells at various time points and concentrations. For post hoc analysis, the Bonferroni correction was used to adjust for multiple comparisons Additionally, Spearman’s rank correlation coefficient was used to evaluate the relationship between *NTN4* expression levels and VAS scores for resting and active pain. To account for potential confounding variables such as age, sex, BMI, and pain scores, ANCOVA was performed. This allowed for the adjustment of these covariates to better assess the relationship between *NTN4* expression and K/L grade. Furthermore, linear regression analysis was conducted to explore the association between *NTN4* expression and key clinical variables, including K/L grade, age, BMI, and VAS scores. A *p*-value less than 0.05 was considered statistically significant.

## Figures and Tables

**Table 1:** Clinical characteristics of knee osteoarthritis patients.

Age (years)	72.9 ± 8.49
Sex, male/female, *n*	20/62
BMI (kg/m^2^)	26.5 ± 4.3
KL grade (2/3/4), *n*	4/21/57
VAS at rest (mm)	3.3 ± 3.1
VAS on movement (mm)	6.6 ± 2.7

**Table 2:** ANCOVA results adjusting for confounders for *NTN4* expression.

Variable	F Value	*p* Value
Age (years)	0.292	0.591
Sex, male/female	3.722	0.058
BMI (kg/m^2^)	2.077	0.154
KL grade	3.804	0.027
VAS at rest (mm)	0.310	0.580
VAS on movement (mm)	0.068	0.795

**Table 3:** Linear regression results for *NTN4* expression variable.

Variable	β	*p* Value
Age (years)	−0.059	0.591
Sex, male/female	−0.211	0.057
BMI (kg/m^2^)	0.156	0.147
KL grade	0.309	0.007
VAS at rest (mm)	−0.066	0.586
VAS on movement (mm)	−0.030	0.805

**Table 4:** Differentially expressed genes between vehicle- and netrin-4 treated cells.

	Sample 1		Sample 2	
	Log2 FC	*p* Value	Log2 FC	*p* Value
*C3*	1.14	5.12 × 10^−55^	1.17	1.86 × 10^−71^
*CXCL1*	1.19	7.43 × 10^−33^	1.06	8.22 × 10^−16^
*CXCL6*	1.16	1.85 × 10^−40^	1.47	3.16 × 10^−96^
*CXCL8*	1.07	5.49 × 10^−16^	5.04	2.29 × 10^−9^
*IL6*	3.77	5.41 × 10^−10^	3.37	4.51 × 10^−7^
*MMP1*	1.07	9.45 × 10^−151^	1.23	1.85 × 10^−17^
*VCAM1*	1.10	1.41 × 10^−60^	1.43	7.16 × 10^−59^
*ENSG00000264668*	2.43	1.06 × 10^−12^	11.15	2.53 × 10^−7^

**Table 5:** Primer sequences.

Gene		Sequence	bp
*C3*	F	ACAAGCTCTGCCGTGATGAA	154
	R	AGCTGAACCTTGACCAGTCG	
*CXCL1*	F	GCTTGCCTCAATCCTGCATC	73
	R	AGTTGGATTTGTCACTGTTCAGC	
*CXCL6*	F	GGTCCTTCGGGCTCCTTGTG	125
	R	ACGCGTAAACAAGTGCAACG	
*CXCL8*	F	ACACTGCGCCAACACAGAAA	89
	R	CAACCCTCTGCACCCAGTTT	
*IL6*	F	GAGGAGACTTGCCTGGTGAAA	199
	R	TGGCATTTGTGGTTGGGTCA	
*MMP1*	F	ACTTACATCGTGTTGCGGCT	164
	R	CGATGGGCTGGACAGGATTT	
*NTN4*	F	TGTTGTCAAGAAGGGCGCTA	159
	R	ACGCGAAGGTTGGTGATCTT	
*VCAM1*	F	CCATCCACAAAGCTGCAAGA	70
	R	CTGGAGCTGGTAGACCCTCG	

## Data Availability

The entire set of RNA-Seq raw data has been submitted to the DNA Data Bank of Japan (DDBJ) and has been assigned the accession code PRJDB18613 (PSUB023706). The data supporting the results of this study can be obtained upon request from the corresponding author, K.U.
